# Effect of hypertension and medication use regularity on postoperative delirium after maxillofacial tumors radical surgery

**DOI:** 10.18632/oncotarget.28048

**Published:** 2021-08-31

**Authors:** Shuyi Kong, Jing Wang, Hui Xu, Kaiqiang Wang

**Affiliations:** ^1^Department of Pain Management, Shanghai Municipal Hospital of Traditional Chinese Medicine, Shanghai University of Traditional Chinese Medicine, Shanghai 200071, China; ^2^Department of Anesthesiology, Shanghai Ninth People’s Hospital, Shanghai Jiao Tong University School of Medicine, Shanghai 200011, China; ^3^Department of Emergency, The Second Affiliated Hospital of Shandong First Medical University, Shandong 271000, China

**Keywords:** the elderly, oral tumor surgery, hypertension, postoperative delirium

## Abstract

The incidence of postoperative delirium (POD) after maxillofacial tumors radical surgery is relatively high. There are a number of evidences showing the relationship between hypertension and decreased cerebral blood flow, as well as the relationship between cerebral ischemia and postoperative cognitive impairment. However, the impact of hypertension in the process of POD and related mechanisms remain unclear.

This study included 98 elderly patients who underwent maxillofacial tumors radical surgery in our hospital, from June 2020 to December 2020. We collected the general condition of patients and related research factors before surgery, and also collected related intraoperative factors. After that, we would follow up the patients for POD evaluation.

The incidence of POD in the hypertension group was 41%, compared with 12% in the nonhypertension group (*P* < 0.05). The incidence of POD in the irregular medication group was 62%, compared with 26% in the regular medication group (*P* < 0.05). Both hypertension (OR = 2.45, 95% CI = 1.11–5.72) and irregular medication use (OR = 2.35, 95% CI = 0.87–5.69) were independent risk factors for POD after this type of surgery in elderly patients.

Hypertension and medication use regularity are closely related to POD. This may be related to the delayed postoperative response caused by intraoperative cerebral ischemia.

## INTRODUCTION

POD [1] is an acute change in mental status characterized by fluctuating disturbances of consciousness, attention, perception and cognition, and it may lead to higher health-resource costs, mortality, and increased hospital stay time.

The incidence of POD is high in patients undergoing maxillofacial tumor [2] radical surgery, which may be related to the long operation time [3], advanced age, fluctuations in blood pressure [4], large blood loss [5] during the resection of the primary tumor, and tracheotomy [6]. The duration of this kind of surgery always reaches 6 hours or longer, and due to the rich blood flow in the maxillofacial region, cutting the tumor can cause massive blood loss in a short time. Because the surgical site was close to the airway, patients routinely need tracheotomy or retain the tracheal tube to maintain the airway patency. Based on the above evidence, POD is more common in patients after this type of surgery. Research data show that long-term hypertension can cause cerebral ischemia [7] damage, and perioperative cerebral ischemia is also related to postoperative cognitive dysfunction [8]. Then, whether long-term hypertension can affect cerebral hemodynamics and increase the risk of POD? At present, cranial ultrasound [9] is used to detect perioperative cerebral blood flow perfusion, although this is helpful to avoid perioperative cerebral blood flow hypoperfusion, it requires real-time intraoperative detection and has certain requirements for posture and operating conditions. If the perioperative indicators like blood pressure and hypertension can be found to have connections with POD, it will be very helpful for early intervention and prevention. Most of the patients undergoing this type of surgery were elderly people, and many patients had hypertension. We performed a preliminary test, and we found that patients with hypertension were more prone to have POD. Therefore, we performed this trial to investigate the risk of POD in patients with hypertension and irregular use of antihypertensive medication and to find the possible mechanism.

## RESULTS

The study cohort consisted of 98 elderly patients undergoing maxillofacial tumors radical surgery; their mean age was 68 years, and 67% were male. We finally enrolled 64 patients in the hypertension group and 34 patients in the nonhypertension group, and 64 patients with hypertension included 38 people in the regular medication group and 26 people in the irregular medication group.

**Figure 1:**
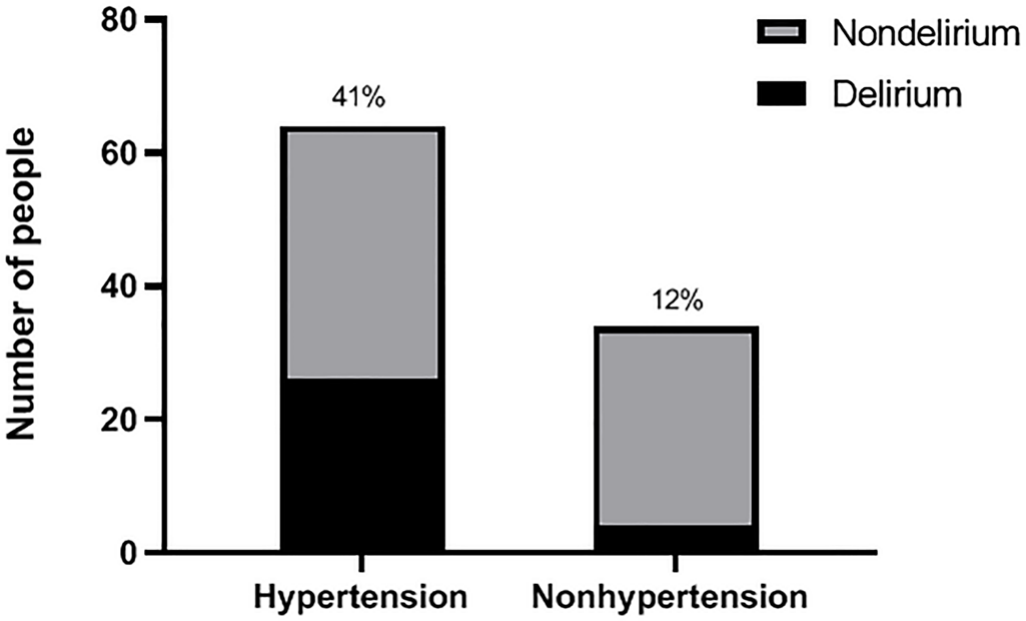
Incidence of POD in hypertension and nonhypertension groups. According to the hypertension assessment scale, the patients were divided into a hypertension group and a nonhypertension group before operation; after operation, they were followed up and then assessed as delirium or nondelirium patients according to the CAM score. The incidence of POD between two groups is shown.

**Figure 2:**
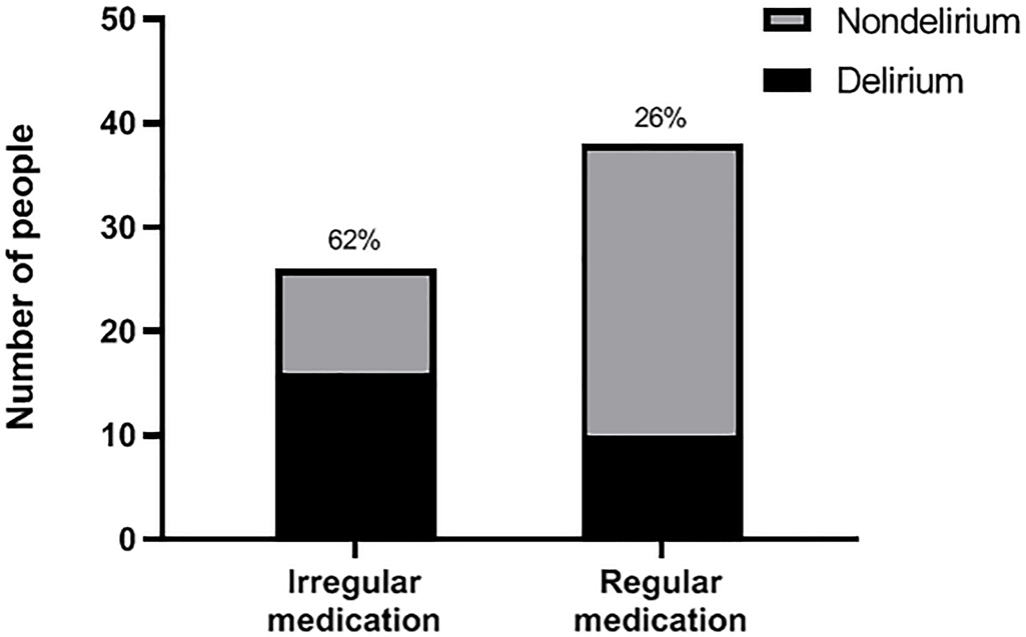
Incidence of POD in irregular medication and regular medication groups. According to the regularity of taking antihypertensive drugs, hypertensive patients were furthur divided into a regular medication group and an irregular medication group before operation; after operation, they were followed up and then classified as delirium or nondelirium patients according to the CAM score. The incidence of POD between two groups is shown.

We made a comparison of the incidence of POD between a hypertension group and a nonhypertension group, as well as between a regular medication group and a irregular medication group, power analysis was shown below. the incidence of POD in the hypertension group was 41% (26 of 64), compared with 12% (4 of 34) in the nonhypertension group (*P* < 0.05, α = 0.05), power (1-β) = 0.89 (Figure 1); the incidence of POD was 62% (16 of 26) in the irregular medication group, compared with 26% (10 of 38) in the regular medication group (*P* < 0.05, α = 0.05), power (1-β) = 0.83 (Figure 2).

As indicated in Table 1 (which illustrate the relative factors of POD in the elderly patients undergoing maxillofacial tumors radical surgery), hypertension, SBP, DBP, and SBP reduction had differences between a delirium group and a nondelirium group (*P* < 0.05); other variables, such as age, BMI, white blood cell count, hemoglobin, surgery duration, blood loss, fentanyl dosage and intraoperative fluid input and tracheotomy number had no difference between a delirium group and a nondelirium group (*P* > 0.05). Logistics regression analysis results (Table 2) showed that hypertension (OR = 2.45 95% CI 1.11–5.72), SBP (OR = 0.91 95% CI = 0.88–0.93), DBP (OR = 0.86 95% CI = 0.79–0.91) and SBP reduction (OR = 0.97 95% CI = 0.95–0.99) were independent risk factors for POD.

**Table 1:** The relative factors of POD in the elderly patients undergoing maxillofacial tumors radical surgery

Characteristic	Delirium (*n* = 30)	Nondelirium (*n* = 68)	*P*
Age, Mean (SD), y	69 (6)	67 (7)	0.31
Sex, No (M,%)	22 (73)	44 (65)	0.40
BMI, Mean (SD), kg/m^2^	23 (3)	22 (4)	0.79
Tracheotomy, No, %	18 (60)	48 (71)	0.30
WBC (×10^9^), Mean (SD)	7.2 (3.0)	6.9 (2.9)	0.63
Hb, Mean (SD), g/L	126 (17)	126 (18)	0.91
Surgery duration, Mean (SD), min	439 (120)	469 (163)	0.36
Blood loss, Mean (SD), ml	567 (222)	529 (232)	0.46
Fentanyl, Mean (SD), mg	0.49 (0.09)	0.48 (0.09)	0.68
Liquid input, Mean (SD), ml	3691 (155)	3660 (132)	0.41
Hypertension, No, %	26 (87)	38 (44)	0.00^*^
Preoperative SBP, Mean (SD), mmHg	153 (15)	134 (12)	0.00^*^
Preoperative DBP, Mean (SD), mmHg	84 (7)	78 (8)	0.00^*^
Intraoperative SBP, Mean (SD), mmHg	115(15)	114(10)	0.46
Intraoperative DBP, Mean (SD), mmHg	52 (7)	53 (6)	0.22
SBP reduction, Mean (SD), mmHg	38 (17)	19 (15)	0.00^*^
DBP reduction, Mean (SD), mmHg	28 (7)	27 (6)	0.51
**Exposure and Covariates for POD in the Hypertension Subgroup**			
**Characteristic**	**Delirium (*n* = 26)**	**Nondelirium (*n* = 38)**	***P***
Age, Mean (SD), y	69 (7)	68 (7)	0.32
Sex, No, (M, %)	20 (77)	30 (79)	0.85
BMI, Mean (SD), kg/m^2^	23 (3)	24 (4)	0.21
Tracheotomy, No, %	14 (54)	26 (68)	0.24
WBC (×10^9^), Mean (SD)	7.8 (2.9)	7.3 (3.6)	0.59
Hb, Mean (SD), g/L	128 (18)	124 (17)	0.44
Surgery duration, Mean (SD), min	443 (128)	467 (207)	0.60
Blood loss, Mean (SD), ml	585 (226)	521 (267)	0.32
Fentanyl, Mean (SD), mg	0.48 (0.08)	0.49 (0.07)	0.57
Liquid input, Mean (SD), ml	3725 (137)	3654 (154)	0.26
Irregular medication, No, %	16 (62)	10 (26)	0.01^*^
Preoperative SBP, Mean (SD), mmHg	153 (15)	134 (12)	0.00^*^
Preoperative DBP, Mean (SD), mmHg	85 (6)	82 (7)	0.00^*^
Intraoperative SBP, Mean (SD), mmHg	116(10)	114(9)	0.33
Intraoperative DBP, Mean (SD), mmHg	51 (8)	52 (6)	0.27
SBP reduction, Mean (SD), mmHg	48 (17)	50 (19)	0.36
DBP reduction, Mean (SD), mmHg	34 (7)	29 (9)	0.00^*^

Abbreviations: SBP/DBP reduction: preoperative SBP/DBP - intraoperative SBP/DBP, BMI: Body Mass Index, WBC: white blood cell, Hb: hemoglobin, SD: standard deviation. ^*^ *P* < 0.05.

**Table 2:** Logistic regression analysis of independent risk factors for POD

Covariate	OR	95% CI of OR	*P*
Hypertension	2.45	(1.11, 5.72)	0.02^*^
SBP	0.91	(0.88, 0.93)	0.00^*^
DBP	0.86	(0.79, 0.91)	0.01^*^
SBP reduction	0.90	(0.85, 0.99)	0.00^*^
**Logistic regression analysis for POD in hypertension subgroup**			
**Covariate**	**OR**	**95% CI of OR**	***P***
Irregular medication	2.35	(0.87, 5.69)	0.02^*^
SBP	0.86	(0.83, 0.90)	0.00^*^
DBP	1.12	(1.03, 1.20)	0.00^*^
DBP reduction	0.88	(0.83, 0.94)	0.00^*^

Abbreviation: CI, confidence interval. ^*^ *P* < 0.05.

We also performed a statistical analysis of variable factors between a delirium group and a nondelirium group in the hypertension subgroup. As indicated in Table 1 (which illustrate the relative factors of POD in the elderly patients undergoing maxillofacial tumors radical surgery), most variables such as age, BMI, white blood cell count, hemoglobin, surgery duration, blood loss, fentanyl dosage and intraoperative fluid input and tracheotomy number had no difference between a delirium group and a nondelirium group (*P* > 0.05) across patients undergoing this kind of operation, while irregular medication use, SBP, DBP, and DBP reduction between the two groups had differences (*P* < 0.05). Logistics regression analysis results (Table 2) showed that irregular medication use (OR = 2.35 95% CI = 0.87–5.69), SBP (OR = 0.86 95% CI = 0.83–0.90), DBP (OR = 1.12 95% CI = 1.03–1.20) and DBP reduction (OR = 0.88 95% CI = 0.83–0.94) were independent risk factors for POD in hypertension subgroup.

## DISCUSSION

### Outcome

Hypertensive patients undergoing this operation under general anesthesia were more likely to have POD than patients with normal blood pressure. For hypertensive patients, the regularity of medication could also have a great impact on the occurrence of POD. We will discuss the main findings of the trial in combination with the the changes in brain hemodynamics among hypertensive patients [10].

### Related mechanism

Scholars has found that cerebral blood perfusion [11] and cerebral blood oxygen saturation are closely related with POD, the regional cerebral blood flow measurements of the frontal, temporal and occipital cortex [12] during delirium are lower than normal state. Cerebral blood flow [13] is tightly controlled via the static and dynamic properties of cerebral autoregulation, this shows that cerebrovascular reactivity can affect the blood supply of the brain, hypertension can reduce the ability of cerebrovascular self-regulation [14], and the changes in vascular stiffness caused by hypertension will further aggravate the decrease in cerebral blood flow [15].

While, in addition to the pathophysiological changes caused by hypertension on the cerebrovascular, anesthesia, controlled hypotension, and insufficient basal blood volume also need to be considered. There have been a number of experimental data that indicate that intraoperative blood pressure fluctuations [16] are related to the occurrence of POD. In our trial, we also analyzed the factors of hypertension and intraoperative blood pressure reduction. We found that delirium and non-delirium patients have significant differences in the number of hypertension and intraoperative blood pressure levels, in order to clarify the most relevant pathogenic factors, we had collected some study results about the effects of long-term hypertension and intraoperative hypotension on POD. According to relevant literature reports, intraoperative hypotension does not directly cause postoperative delirium [17], but for patients with different cerebral blood flow regulation [18], using the same blood pressure maintenance standard will significantly increase the incidence of postoperative POD, which indicates that compared with intraoperative blood pressure, more attention should be paid to the patient’s basic cerebrovascular function and adjustment ability. According to previous research reports, hypertension could impair the cerebrovascular reactivity in older adults, and impaired cerebrovascular reactivity may be a predictor for delayed cerebral ischemia [19]. This may provide a reasonable explanation for delirium that usually occurs 1 to 2 days after surgery rather than immediately.

This hypothesis of POD mechanism related to cerebral ischemia also explains why intraoperative hypotension alone does not cause POD, while excessive intraoperative blood pressure reduction in patients with hypertension will cause POD, which is related to the range of hypotension that can be tolerated by different cerebrovascular functional states. If the cerebrovascular elasticity and adjustment ability are poor, POD is easier to happen.

Based on the relative mechanism and statistical data analysis, we can further confirm that the preoperative history of hypertension and the regularity of drug treatment in hypertensive patients are related to the occurrence of POD. If we evaluate the patient’s preoperative blood pressure and medication use regularity and find that he has a higher risk of POD, preventive psychological and drug intervention [20] will be required.

For anesthesiologists, perioperative blood pressure management should be paid enough attention to avoid the risk of creating hypotension; for patient-in-charge doctors, preoperative evaluation of the possibility of POD can be made based on this, and preoperative prevention [21] should be reasonably adopted; for surgeons, for high-risk patients with POD, the time for resection of the primary focus should be shortened and the requirements for controlled blood pressure should be moderately reduced; for patients with hypertension, long-term blood pressure management is particularly important, regular blood pressure monitoring and regular medication use should be done.

### Limitations

This trial is an observational study, and because the course of hypertension naturally evolves regardless of human intervention, and the formation and development of hypertension may be affected by a combination of drugs and individual adjustments, we cannot interfere with the target variables through an randomized controlled trial to determine the influence of these factors of the outcome indicators, including the correlation between hypertension and low cerebral blood flow. However, we conducted statistical analysis and summarized multiple correlations from clinical research, and we finally confirmed the relevance and rationality of our analysis.

## MATERIALS AND METHODS

### Study design and data source

**Figure 3:**
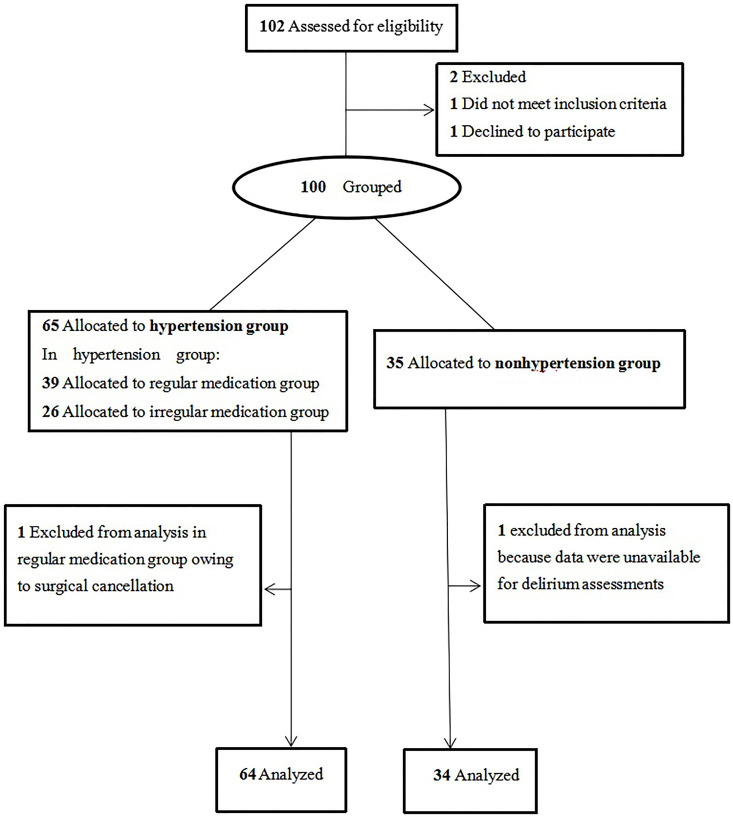
Patient enrollment. This figure shows us the initial screening number of cases, the number of excluded cases that do not meet the criteria, and the number of final included cases in hypertension group and nonhypertension group.

We performed a nested case-control study of patients 60 years or older who underwent maxillofacial tumors radical surgery with some data retrieved from the Medical Record Management System of Shanghai Ninth People’s Hospital Affiliated with Shanghai Jiaotong University School of Medicine and some data collected from the trial process from June 14, 2020, to December 7, 2020. This study followed the STROBE Statement reporting guidelines. A patient inclusion flow diagram was shown in Figure 3. Research Ethics Committee approval was obtained, and a written informed consent was completed for each enrolled patient. Registration of their clinical trial occurred prior to the start of the trial and any patient enrollment undertaken. The registration number was NCT04433416, the principal investigator’s name was Lei Zhang, the date of registration was June 13, 2020.

Inclusion criteria were age 60 years or older, proposed maxillofacial tumors radical surgery (with maxillofacial free flap transplantation), and ASA classification I-II. The exclusion criteria were preoperative cognitive dysfunction (Abnormal MMSE score [22], mental disease [23], dementia [24]), severe anemia before surgery (Hb < 80 g/L), preoperative cerebral infarction or cerebral hemorrhage, long-term use of sedative or analgesic drugs, and unwillingness to cooperate with the research.

The Medical Record Management System contained all medical and pharmacy claims records of inpatients, and from this system, we could collect the patients’ demographic information, relevant systemic disease history and information about doctors’ orders related to medical procedures.

### Patient data collection

**Figure 4:**
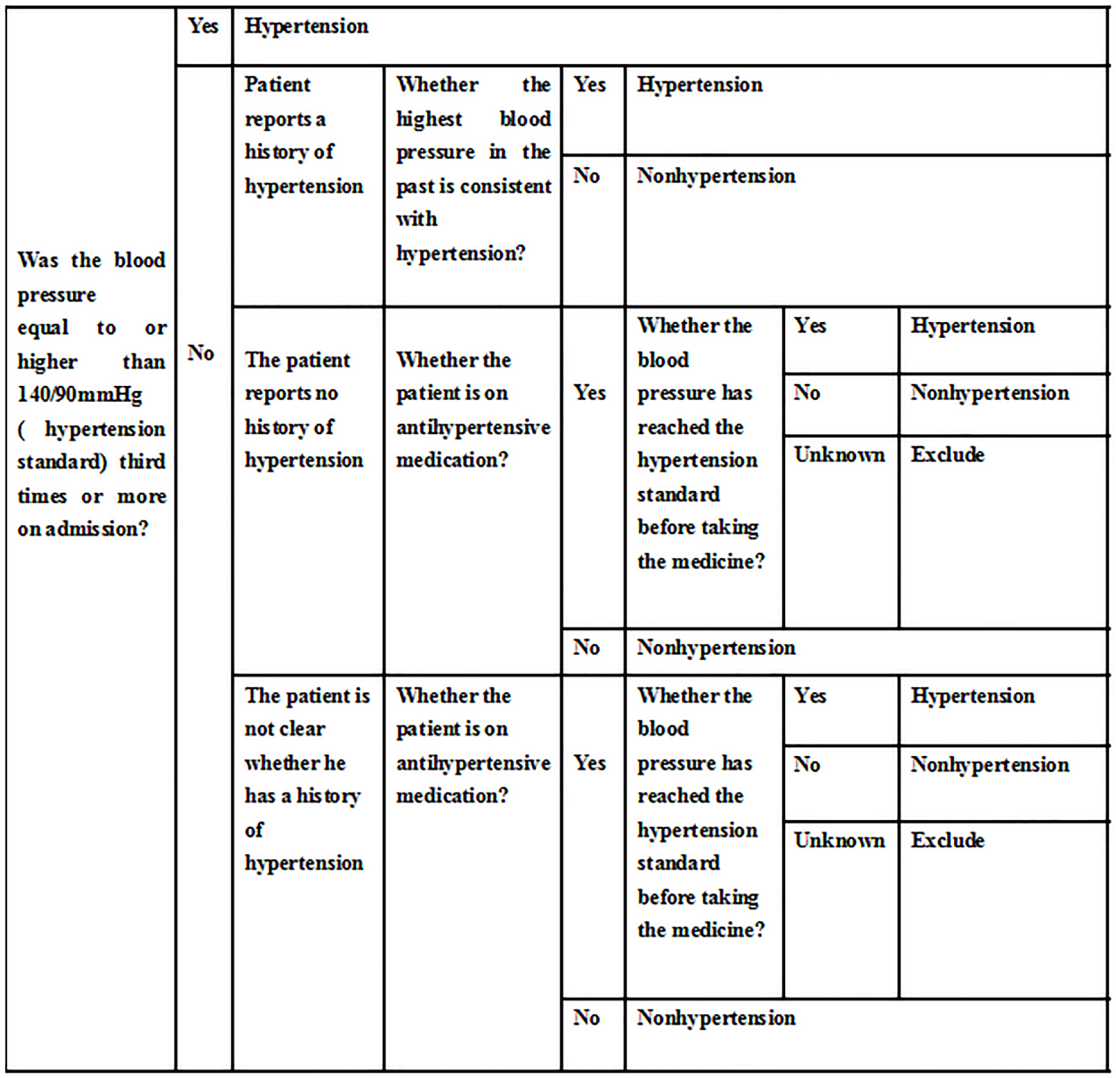
Hypertension assessment. This figure provides a detailed description of the diagnosis of hypertension in the enrolled patients.

The researchers conducted preoperative visits with the patients 1–2 days before the operation and collected data on the preoperative conditions of the patients (evaluation indicators of anesthesia, past disease history, surgical history, demographic data, etc.). For patients who met the inclusion criteria, we used a cuff blood pressure monitor to measure the patient’s preoperative blood pressure in a calm state at different time periods, and according to the hypertension assessment scale (Figure 4), we divided patients into a hypertension group and a nonhypertension group. For patients in the hypertension group, they were further divided into a regular medication group and an irregular medication group (regular medication was defined as long-term hypertension drug treatment and taking the medication at the prescribed time and dose; irregular medication referred to noncompliance with regular medication standards, including temporary medication due to high blood pressure before surgery and medication only when blood pressure was increased in daily life).

### Perioperative management and perioperative index collection

The anesthesia process for each patient was completed by a randomly assigned anesthesiologist, and the investigator did not perform any intervention during the perioperative period. After the patient entered the operating room, routine vital sign monitoring (ECG, noninvasive blood pressure, blood oxygen saturation) was performed, and the amount of fluid lost according to the preoperative fasting time was replenished. After the induction of anesthesia, arterial puncture and deep venous puncture were conducted. During the operation, two-way venous access was used to infuse fluids. When the amount of bleeding was heavy, the speed of infusion was accelerated, and the urine output was kept within the normal range. General anesthesia was induced with midazolam (0.02–0.04 mg/kg), fentanyl (2 μg/kg), propofol (2.0–2.5 mg/kg), and cisatracurium besylate (0.2 mg/kg) and maintained with remifentanil (0.1–0.2 μg/kg/min), 1% propofol (2–5 mg/kg/h), and isoflurane. Partial pressure of carbon dioxide was maintained between 35 and 45 mmHg. We implemented intraoperative rectal temperature monitoring and we used a thermometer or heating blanket to maintain intraoperative body temperature between 35°C and 37°C.

The researchers collected data on intraoperative blood loss, operation time, fentanyl dosage, intraoperative fluid infusion and the invasive arterial pressure value (every 5 minutes) based on the anesthesia record sheet. After the operation, the patient entered the intensive care unit with a tracheotomy or a tracheal tube in place to maintain the patency of the airway. After one night of monitoring in the intensive care unit, the patient would be transferred to the general ward on the first day after surgery. POD was assessed at general ward at 8–10 pm every night from the first day the patient returned to the ward to the third day using the Confusion Assessment Method (CAM) [25]. The CAM assessment [26] was performed by the main researcher and included a structured cognitive examination.

### Case identification

We identified SBP (systolic blood pressure) reduction, DBP (diastolic blood pressure) reduction as the preoperative SBP, DBP value minus the intraoperative SBP, DBP value. All blood pressure data were the mean values of the data collected.

The evaluation criteria in this hypertension assessment scale (Figure 4) strictly complied with the 2016 Hypertension Diagnostic Guidelines [27]. We summarized different situations and applicable conditions based on the guidelines for hypertension management and related literature reports, and made this scale. These included two situations where antihypertensive drugs had been taken and antihypertensive drugs had not been taken: If the patient had not taken antihypertensive drugs on the day of blood pressure measurement, the actual measurement value would be evaluated; if the patient had taken antihypertensive drugs on the day of blood pressure measurement, then, the blood pressure value of the patient when the patient had not taken the medicine was obtained as a diagnostic basis by asking. If the patient was not aware of his previous blood pressure and had taken antihypertensive drugs due to high blood pressure after admission, the diagnosis could not be confirmed and the case would not be included.

### Exposure

We examined hypertension, medication use regularity as the main research factors of POD in our research. We had to strictly measure the preoperative blood pressure to determine the baseline blood pressure level and collect the blood pressure data in the anesthesia record sheet to calculate the perioperative blood pressure decline value.

Other influencing factors for POD were also considered, such as age, surgery duration, blood loss, white blood cell count, hemoglobin, tracheotomy, intraoperative fentanyl dosage and fluid management, preoperative blood pressure and intraoperative blood pressure reduction.

### Statistical analysis

The main results of the comparison of POD incidence between a hypertension group and a nonhypertension group, as well as between a regular group and a irregular group were analyzed with χ^2^ tests, and the power value was also calculated.

Patient related data and surgical characteristics were analyzed between a delirium group and a nondelirium group by *t* tests, Fisher exact tests and χ^2^ tests. The counting data like hypertension number, irregular medication number, sex number and tracheotomy number were analyzed by χ^2^ tests or Fisher exact tests. Measurement data such as blood pressure, BMI, Hb, WBC, SBP, DBP, DBP reduction and SBP reduction were in accordance with the normal distribution, and they were analyzed by *T* tests. The statistical analysis of the data in the hypertension subgroup were all the same.

Analyses were conducted using SPSS 20.0 with 2-sided hypothesis testing (α = 0.05), and *P* < 0.05 was considered significant. A reliable theory [28] (name: Applied logistic regression) that using a significance level as high as 0.2 as a screening criterion for logistics regression’ variable selection had been widely used (cited by many magazines like NEJM, JCO). Therefore, we included data that meets *P* < 0.2 into logistics regression analysis. Finally, to find out independent risk factors of POD, we put hypertension, SBP, DBP, and SBP reduction into a logistics regression analysis; and in the hypertension subgroup, we put irregular medication use, SBP, DBP, and DBP reduction into a logistics regression.

## Abbreviations

POD: postoperative delirium
MMSE: Mini-mental State Examination
SBP: systolic blood pressure
DBP: diastolic blood pressure
MAP: mean arterial pressure
Hb: hemoglobin
BMI: Body Mass Index
WBC: white blood cell
CI: confidence interval
SD: standard deviation
CAM: Confusion Assessment Method
